# Glottal Area Waveform Measurements for Healthy Female and Male Speakers in Typical, High-Frequency, and Soft Phonation

**DOI:** 10.1044/2026_JSLHR-25-00611

**Published:** 2026-04-16

**Authors:** Rita R. Patel, Zhaoyan Zhang, Michael Döllinger, Andrew Adeola, Stefan Kniesburges

**Affiliations:** a Department of Speech, Language and Hearing Sciences, Indiana University Bloomington; b Department of Head and Neck Surgery, University of California, Los Angeles; c Division of Phoniatrics and Pediatric Audiology, Department of Otorhinolaryngology—Head and Neck Surgery, University Hospital Erlangen, Medical School, Friedrich-Alexander University Erlangen–Nürnberg, Germany; d Indiana University School of Medicine, Indianapolis

## Abstract

**Purpose:**

This study aimed to examine vocal fold kinematic characteristics associated with typical-frequency and vocal-intensity, high-frequency, and soft-intensity phonation in vocally healthy adults.

**Method:**

Glottal area waveform (GAW) was measured from high-speed videoendoscopy in a total of 66 adults (41 women and 25 men) during sustained /i:/ production across the three tasks, resulting in a total of 594 phonations. Statistical analysis of glottal cycle quotients (open quotient [OQ], speed quotient [SQ], rate quotient [RQ], glottal gap index [GGI]), glottal cycle periodicity (amplitude, time periodicity [TP]), glottal cycle symmetry (phase asymmetry index, spatial symmetry index, amplitude symmetry index), normalized maximum area declination rate (MADRn), and amplitude-to-length ratio (ALR) was conducted. Principal component analysis was used to identify laryngeal strategies underlying the three tasks.

**Results:**

High frequency and soft intensity resulted in changes in SQ, RQ, MADRn, and ALR in female participants, whereas in male participants, they impacted OQ, RQ, GGI, TP, MADRn, and ALR. High-frequency phonation is primarily achieved through increased cricothyroid muscle activity, while soft intensity is primarily achieved by reduced vocal fold adduction and subglottal pressure with compensatory cricothyroid activation.

**Conclusion:**

High-frequency and soft-intensity phonations involve distinct laryngeal adjustments and clinically measurable, sex-dependent changes in GAW, highlighting the need to tailor voice therapy to physiological strategies and sex-based differences.

The assessment of dysphonia involves a careful evaluation of the subsystems of voice production, particularly of the vocal folds. Direct evaluation of the structure and vibratory dynamics through laryngeal imaging is central to the clinical evaluation of organic and functional voice disorders, as it offers insights into underlying mechanisms that noninvasive techniques such as electroglottography or auditory perceptual evaluation cannot provide (Herbst et al., 2011, 2014; Laukkanen et al., 2007; Švec et al., 1996). The major aim of visualizing the vibrating vocal fold through laryngeal imaging techniques is to assist with finding the appropriate cause of dysphonia and measuring treatment outcomes by evaluating disturbances in the structure and the resulting vibratory motion. Toward this goal, normative data of vocal fold vibration across varying vocal frequencies and intensities are required.

Stroboscopy, the current clinical standard (Schwartz et al., 2009), provides phase-averaged vibratory patterns, but it lacks the ability to capture cycle-to-cycle motion (Deliyski & Hillman, 2010; R. R. Patel et al., 2008). High-speed videoendoscopy (HSV), with its higher temporal resolution, has proven superior for evaluating cycle-to-cycle variations in vibratory motion (Olthoff et al., 2007; R. R. Patel et al., 2008, 2011, 2012; Timcke et al., 1958; Zacharias et al., 2016). Despite HSV's advantages, its clinical use remains limited, largely due to a lack of quantitative data across varying frequencies and vocal intensities. Another limitation is the inability to process data within a time frame compatible with routine clinical care. However, the addition of simultaneous audio recording allows clinicians to rapidly navigate to events of interest and apply a previously developed HSV visual rating form, called voice-vibratory assessment with laryngeal imaging (Poburka et al., 2017), thereby expediting the analysis and aligning HSV assessment more closely with established stroboscopic rating practices.

Although the need for objective assessment of vocal fold vibrations across varying phonatory conditions has been established for videostroboscopy (Woo, 1996), similar data using HSV are lacking, particularly from large samples of vocally healthy speakers. Previous high-speed laryngeal imaging studies have predominantly concentrated on the quantification of vibratory dynamics during typical (normal) phonation (Ahmad et al., 2012; Arias-Vergara et al., 2023; Bohr et al., 2013, 2014; Inwald et al., 2011; Lohscheller et al., 2013; R. R. Patel et al., 2014a, 2014b, 2021, 2023; Schlegel et al., 2020; Schraut et al., 2025; Yamauchi et al., 2014, 2016). To interpret vocal function across the range of voice disorders, data collected in a large range of normal tasks with varying vocal frequency and intensity are needed with HSV. Currently, the influence of fundamental frequency (*F*0) and vocal intensity on the shape of the glottal area waveform has only been investigated in a small number of participants using HSV. In the classic studies by Timcke et al. (1958, 1959), glottal cycle quotients of open quotient (OQ) and speed quotient (SQ) were elaborately investigated across variations in pitch and loudness using high-speed films in two male participants. More recently, R. R. Patel et al. (2022) investigated the relationship between airflow, subglottal pressure, and glottal area waveform in 13 vocally healthy adults (women: 6, men: 7) across typical, loud, and soft conditions, under various phonation types including breathy, flow, neutral, and pressed. We were unable to locate any other in vivo studies in the literature that quantitatively evaluated changes in multidimensional glottal area waveform comprehensively as a function of increased *F*0 and soft vocal intensity using HSV on a large number of subjects, particularly with limited investigation at soft vocal intensities. Collecting objective multidimensional HSV measurements in vocally healthy individuals under different phonatory conditions is a critical first step toward understanding abnormal vocal function in both clinical and research contexts. These foundational data also have the potential to pave the way for integrating state-of-the-art machine learning models into the diagnosis and management of voice disorders.

Normal vocal fold vibratory motion is complex and depends on the combination of anatomical and physiological factors, as well as vocal frequency and intensity. High–fundamental-frequency phonations assess the upper limits of vocal fold elongation, whereas soft phonation evaluates the ability to maintain adequate vocal fold adduction. Both tasks are routinely recommended protocols in the clinic to evaluate the integrity of the system. Hence, in this study, we aim to establish quantitative group data of changes in the glottal area waveform obtained from HSV for three voice tasks: typical pitch and loudness, high pitch and typical loudness, and soft voice and typical pitch. Typical pitch and loudness (also referred to as habitual, modal, normophonic, comfortable, neutral phonation) in this study was defined as the attractor state pitch and loudness used by participants in daily conversations. We focus on clinically relevant measures of vocal fold vibratory function derived from the entire glottal area waveform, as it provides a more complete picture of the vibratory motion along the entire margin of the vocal fold (Herbst et al., 2014; Kist et al., 2021; R. R. Patel et al., 2014a). Although the vocal fold vibration amplitude is important in determining the produced vocal intensity, the glottal closure pattern, including the closing speed and duration of glottal closure, is more important in determining the produced voice quality (Kreiman et al., 2014). Thus, in this study, we focus on vibratory measures of both the vibration amplitude and the glottal closure pattern, including measures of OQs, SQ, minimal glottal opening area, normalized maximum glottal area declination rate, and amplitude-to-length ratio (ALR). We also include measures of periodicity and asymmetry in vocal fold vibration to identify acceptable degrees of asymmetry and aperiodicity in normal-sounding voices.

In general, *F*0 increase is achieved primarily through an increased activation of the cricothyroid muscles (Hirano, 1988; Hirano et al., 1969), which elongates and stiffens the vocal folds. Thus, increasing *F*0 is likely to result in a reduced vocal fold vibration amplitude and a reduced ALR. Increased cricothyroid activation also often reduces vocal fold thickness and vocal fold approximation, both of which increase the OQ and reduce the skewedness of the glottal area waveform in ex vivo experiments (Choi et al., 1993; Mendelsohn et al., 2015; Zhang, 2016b). The reduced vocal fold thickness and approximation may also increase the minimal glottal opening area, particularly in males where the relatively longer larynges are more easily pushed open by the subglottal pressure (Zhang, 2021b). *F*0 can also be increased by increasing vocal fold approximation or increasing subglottal pressure (Titze, 1989a; Van Den Berg & Tan, 1959; Zhang, 2016b). Increasing vocal fold approximation often decreases the minimal glottal opening area, vibration amplitude, and ALR and may also slightly reduce the OQ. Increasing subglottal pressure alone, on the other hand, often increases the vibration amplitude, minimal glottal opening area, and ALR, but it often has small effects on the OQ or the skewedness of the glottal area waveform (Zhang, 2021a, 2024a).

Whereas vocal intensity is primarily determined by subglottal pressure, laryngeal and vocal tract adjustments also play an important role in vocal intensity and loudness control (Herbst & Story, 2022; Titze, 1992, 2023; Titze & Sundberg, 1992; Zhang, 2016b, 2025), particularly for voices of soft-to-moderate vocal intensity (Hirano et al., 1969). Although speakers often make simultaneous adjustments in the three subsystems (Holmberg et al., 1988; Isshiki, 1964; S. Patel et al., 2011; Stathopoulos & Sapienza, 1993; Zhang, 2024b), individual speakers may adopt different strategies and may rely on the adjustment of one subsystem more than the other two. Thus, interspeaker variability is expected when comparing vocal fold vibration between typical vocal-intensity and soft–vocal-intensity conditions. For speakers who rely more on lowering the subglottal pressure to produce a soft voice, we would expect a reduced vocal fold vibration amplitude, resulting in a reduced ALR compared with the typical loudness condition. For speakers relying more on laryngeal adjustments, soft voices can be produced by a reduced degree of vocal fold adduction, which would result in an increased minimal glottal opening area and OQ, as well as a less skewed glottal area waveform. With the reduced vocal fold approximation, we also expect the glottal area waveform to be more similar between male and female voices. Reducing vocal intensity can also be produced by a reduced resonance effect of the vocal tract, in which case changes in vocal fold vibration are expected to be small, considering the weak source–tract interaction in the pitch range typical of speech (Zhang, 2023). We also expect relatively higher cycle-to-cycle variation in both amplitude and period in soft voice, as it is being closer to phonation onset.

In addition to establishing quantitative group data for the bias-free clinical identification of voice disorders and the quantification of treatment outcomes, detailed measurements of the glottal area waveform changes due to high frequency and soft vocal intensity would (a) facilitate the development of advanced voice production inversion systems (Wurzbacher et al., 2006), which may provide insights into the underlying laryngeal adjustments made to vary frequency and vocal intensity, and (b) allow for detailed insights into the source–filter coupling and the effects of the vocal fold dynamics on the acoustic output. Hence, in this study, we examine the following questions: (a) What are the normative values of glottal area waveform measures across men and women at typical pitch and loudness, and how are they affected when producing high-frequency phonation and soft phonation? (b) Which glottal area waveform measures account for the differences in the kinematic space between typical phonation, high-frequency phonation, and soft–vocal-intensity phonation across men and women?

## Method

### Data Collection

A total of 92 vocally normal, young adults were recruited at the Vocal Physiology and Imaging Laboratory at the University of Kentucky and at Indiana University after signing the institutional review board–approved (08-0995-P3H/1210009782) consent forms. Participants were recruited for the study if they were perceptually judged to have normal voice quality on the Grade, Roughness, Breathiness, Asthenia, and Strain scale (Hirano, 1981) by a speech-language pathologist specialized in the area of voice. Additionally, the participants did not have a history of smoking, were not professional singers, and did not have vocal fold pathologies. Due to heightened gag reflex, 22 participants could not complete all three tasks. Hence, simultaneous HSV and acoustic recordings were obtained from 70 participants.

High-speed videoendoscopic recordings were captured at 4,000 frames per second with a spatial resolution of 512 × 256 pixels, for a duration of 4.094 s, using a high-speed video camera (PENTAX Medical Photron FASTCAM MC2.1) for steady-state phonation of the vowel /i:/ at different pitch and loudness levels. The vowel /i:/ was selected because it facilitates clear endoscopic visualization of the vocal folds by elevating the larynx and inducing anterior–superior displacement of the epiglottis, thereby clearing the line of sight. Acoustic signals were recorded simultaneously at a sampling frequency of 50 kHz to verify the presence of steady-state phonation and to obtain vocal frequency and intensity. The manufacturer-provided lavalier microphone (Audio-Technica ASP-0091, PENTAX Medical Model No. 7175-6,000) was used, with a fixed mouth-to-microphone distance at 5 in. (12.7 cm) maintained by securing the microphone to the participants' clothing using a clip. Participants were instructed to phonate sustained /i:/ at self-selected typical normal pitch and normal loudness (NPNL), high pitch and normal loudness (HPNL), and soft voice with typical pitch (NPS). Self-selected steady-state phonation was chosen, compared with a target pitch and loudness level, to minimize the effects of increased effort during the targeted production of pitch and loudness (Hanson et al., 1990). This approach not only reflects typical clinical practice but also enhances clinical validity by capturing more natural phonatory behavior, providing a realistic representation of the participants' voice use. Vocal frequency and intensity manipulations were used to reveal the range of vocal fold vibratory behavior that may not be evident during typical phonation. All trials began at participants' self-selected habitual speaking frequency and vocal intensity level. Participants were then required to produce high-frequency phonation, followed by soft phonation. Recordings were retained only if they met the auditory–perceptual quality criteria, including stable phonation and adequate visualization of the vocal folds. Three trials of each condition were performed for the three tasks without any vocal warm-up. Hence, a total of nine video recordings were included for each participant. Data collection lasted for approximately 30 min for each participant. Due to the lack of usable audio signal from four participants (three women, one man), only data from 66 participants (41 women [26.83 ± 5.47 years old], 25 men [27.96 ± 5.19 years old]) were subjected to further analysis. Overall, 594 phonations from 66 participants were analyzed in this study. An a priori power analysis was not conducted for this portion of the study due to the exploratory nature.

### Data Segmentation

Prior to analysis, the high-speed videos were examined for steady state based on the waveform and the narrow band spectrogram of the acoustic signal. Additionally, the video segments with minimal vertical and horizontal motion of camera focus over the duration of 100 cycles, as determined by human visual inspection, were selected for segmentation. The duration of 100 cycles of steady-state phonation was considered a reasonable compromise to the maximum sample size that could be obtained for reliable and valid measurements with minimal influence from endoscopic motion (Schlegel et al., 2018). Video segmentation and computation of the kinematic measures were performed using the software application Glottis Analysis Tool (Version 2018) developed at the Division of Phoniatrics and Pediatric Audiology, University Hospital Erlangen. The Glottis Analysis Tool was used due to its robustness and global accessibility as freely available software (Kist et al., 2021). It is currently utilized by 35 universities across 15 countries, highlighting its broad adoption and reliability in voice research and clinical applications.

### Data Analysis

A total of 11 kinematic measures representing different components of the glottal dynamics were investigated: glottal area quotients (reflecting the opening and closing processes), glottal cycle-to-cycle periodicity, glottal cycle symmetry (timing, similarity in size, and absolute size differences), glottal area derivatives, and glottal mechanical measures. The goal was to analyze the influence of the phonatory task and gender on the different components of the glottal area waveform: shape, symmetry, periodicity, and mechanical factors. The following glottal area waveform measures were extracted: (a) glottal cycle quotients, including OQ, SQ, rate quotient (RQ), and glottal gap index (GGI); (b) glottal cycle periodicity, including amplitude periodicity (AP) and time periodicity (TP); (c) glottal cycle symmetry, including phase asymmetry index, spatial symmetry index, and amplitude symmetry index; (d) glottal area waveform derivatives, including normalized maximum area declination rate (MADRn) by the product of peak-to-peak amplitude and *F*0 of the glottal area waveform to isolate changes in waveform shape from changes in amplitude or frequency (Titze & Worley, 2009; Zhang, 2021b); and (e) ALR (see Table 1). The glottal area waveform measures were computed over each cycle and subsequently averaged over the 100 segmented cycles. The detailed computations of these measures are listed in Table 1 and also available in Kist et al.'s (2021) study.

**Table 1.** List of glottal area waveform kinematic measures derived from high-speed videoendoscopy (HSV) during sustained phonation.

HSV measures	Description and interpretation
(A) Glottal cycle quotients	
Open quotient (range: 0–1)	Duration of the open phase of a glottal cycle divided by the entire cycle duration. The value of 1 is an incomplete glottal closure. The higher the value, the shorter the closed phase.
Speed quotient (range: 0–∞)	Ratio of the opening and closing duration of a glottal cycle. The value of 1 indicates that the opening and closing parts of the glottal cycle are of equal duration. Values < 1 indicate that the opening process is shorter, and values > 1 indicate that the opening phase is longer than the closing phase.
Rate quotient (range: 0–∞)	Time when the glottis is entirely closed plus time of glottis opening process divided by time interval of the closing process of a glottal cycle. The value of 1 indicates that the sum of opening and closed phases is of equal length compared with the closing phase of the glottal cycle. The lower the value, the slower the glottis closes.
Glottal gap index (range: 0–1)	Minimum glottal area of a glottal cycle divided by the maximum glottal area. The value of 0 indicates complete closure, and the higher the value, the larger the glottal gap in relation to the entire open glottis.
(B) Glottal cycle periodicity	
Amplitude periodicity (range: 0–1)	Average ratio of the smaller to the larger amplitude between two successive cycles. The higher the value, the higher the periodicity and smaller the differences in dynamic range between neighboring cycles.
Time periodicity (range: 0–1)	Ratio of the shorter to the longer duration between two successive cycles. The higher the value, the higher the periodicity and smaller the differences in cycle lengths between neighboring cycles.
(C) Glottal cycle symmetry	
Phase asymmetry index (range: 0–1)	Normalized time difference between the maximum glottal area of the left and right vocal folds within each cycle. A large value indicates a greater phase shift between the left and right glottal area waveforms.
Spatial symmetry index (range: 0–1)	The absolute difference in area between the left and right partial glottal area waveforms during one cycle, with higher values indicating greater asymmetry in size/area between two partial glottal area waveforms.
Amplitude symmetry index (range: 0–1)	Minimum value of the maximum glottal area between the left and right vocal folds divided by the maximum of both areas. The higher the value, the higher the left and right glottal area waveform symmetry.
(D) Glottal area waveform derivatives	
Normalized maximum area declination rate (range: 1–∞)	Maximum rate of declination of the glottal area waveform in the closing phase normalized by the product of the fundamental frequency, pi, and peak-to-peak amplitude of the glottal area waveform. As the value approaches 1, the waveform would be sinusoidal.
Amplitude-to-length ratio (range: 0–∞)	Ratio between the dynamic glottal area and the length of the maximal open glottis. The smaller the value for a constant glottal length, the stiffer the vocal fold tissue or the narrower the glottis.

### Acoustic Analyses

The microphone was calibrated by measuring sound pressure level (SPL) from sustained phonation at a 30-cm mouth-to-microphone distance using a sound-level meter (Quest) with C-weighting and slow response (R. R. Patel et al., 2018). Acoustic analysis of vocal frequency and SPL was obtained using the Praat software (Boersma, 2001).

### Statistical Analyses

Statistical analysis was performed to examine, first, the extent to which the kinematic features derived from the glottal area waveform were different across typical phonation, high pitch, and soft for male and female participants. The Shapiro–Wilk test was performed to assess the normality of the 11 glottal area waveform measures, including vocal intensity and *F*0. Values of the dependent variables were averaged across the three trials for the NPNL, HPNL, and NPS conditions. The Shapiro–Wilk test revealed that SPL (dBC) and ALR were normally distributed (*p* > .05) for female participants, whereas only one parameter, SPL (dBC), was normally distributed for male participants (see Table 2). Comparisons across the three groups (NPNL, HPNL, and NPS) were performed using analysis of variance for normally distributed measures and the Kruskal*–*Wallis *H* test for nonnormally distributed measures. For significant group findings across normally distributed measures, post hoc tests were performed using the least significant difference tests. Significant group findings for nonnormally distributed measures were subjected to the Mann–Whitney *U* test, with post hoc tests adjusted using Bonferroni correction (*p* ≤ .05/10 for female participants and *p* ≤ .05/11 for male participants). Effect sizes were calculated using Cohen's *d* for the comparison of means. Statistical analysis was performed using IBM SPSS Statistics, Version 21.0.

**Table 2.** Results of the Shapiro–Wilk test for normality in females (*n* = 41) and males (*n* = 25).

Measures	Overall, *M* ± *SD* values		Shapiro–Wilk statistic		*p* value	
	Female	Male	Female (*df* = 41)	Male (*df* = 25)	Female	Male
Open quotient	0.95 ± 0.09	0.84 ± 0.17	.65	.85	.000	.000
Speed quotient	1.71 ± 0.43	1.40 ± 0.47	.94	.92	.000	.000
Rate quotient	1.92 ± 0.68	2.09 ± 1.17	.81	.84	.000	.000
Glottal gap index	0.09 ± 0.09	0.03 ± 0.06	.88	.62	.000	.000
Amplitude periodicity	0.98 ± 0.00	0.99 ± 0.01	.94	.89	.000	.000
Time periodicity	0.96 ± 0.01	0.97 ± 0.02	.97	.85	.007	.000
Phase asymmetry index	0.07 ± 0.04	0.06 ± 0.04	.91	.91	.000	.001
Spatial symmetry index	0.14 ± 0.08	0.15 ± 0.10	.85	.86	.000	.000
Amplitude symmetry index	0.81 ± 0.09	0.81 ± 0.11	.90	.89	.000	.016
Normalized maximum area declination rate	1.49 ± 0.46	1.39 ± 0.35	.87	.96	.000	.022
Amplitude-to-length ratio	11.10 ± 3.14	13.15 ± 4.17	.98	.96	**.092**	.013
Intensity SPL (dB)	62.3 ± 5.7	60.1 ± 6.7	.99	.98	**.375**	**.165**
*F*0 (Hz)	261 ± 63	168 ± 55	.92	.84	.000	.000

*Note.* Normally distributed measures (*p* > .05) are highlighted in bold. The means and standard deviations across all three tasks are provided. Statistically significant values are in bold. *F*0 = fundamental frequency.

Principal component analysis (PCA) was conducted to evaluate which glottal area waveform measures account for the differences in the kinematic space between typical phonation, high-frequency phonation, and soft phonation and to identify strategies used by the speakers to produce different speech tasks. Because of the high multicollinearity between the different measures, only a selected subset of measures was included in the PCA to facilitate interpretation. The measures were selected so that the variance inflation factors were below 5 between the selected measures. These measures include the SPL, *F*0, OQ, GGI, ALR, and MADRn. The inclusion of both the two control measures (SPL and *F*0) and four measures of the glottal area waveform allowed us to relate changes in the glottal area waveform measures to changes in the target SPL and *F*0, thus revealing strategies used by speakers to achieve the intended changes in target SPL and *F*0. For each participant and speech task, the corresponding values of the six measures were collected into a column vector. The vectors from all participants and speech tasks were then assembled into a 6 × 198 matrix, on which the PCA was conducted in MATLAB (Version 2019b; MathWorks, Inc.). Differences between male and female participants, along the first two principal components (PC1 and PC2), were assessed using two-sample *t* tests. Within each participant, changes in principal component coefficients between speech tasks (HPNL–NPNL for increased pitch and NPS–NPNL for reduced loudness) were computed, and one-sample *t* tests were used to determine whether these within-subject changes in PC1 and PC2 differed significantly from zero for male and female participants.

## Results

### Task Performance

Before subjecting the data to statistical analyses across the glottal area waveform measures, all participants were statistically evaluated for adequacy of performance across frequency and vocal intensity conditions, based on the distribution of the data (see Table 2). For both female and male participants (see Table 3), expected statistical differences in *F*0 and SPL (dBC) were obtained. That is, *F*0 for the high-pitch task was higher than the typical phonation with similar and softer vocal intensity (see Table 4). The difference in *F*0 was not statistically significant for NPS versus NPNL conditions. Similarly, for vocal intensity condition, the SPL was lower for soft voice, followed by typical phonation (see Table 4), and the difference was not statistically significant between the NPNL and HPNL conditions. In summary, the data verified that participants graded the vocal frequency and intensity of their phonation.

**Table 3.** Comparison between normal pitch and normal loudness (NPNL), high pitch and normal loudness (HPNL), and normal pitch and soft (NPS) across the variables of sound pressure level (SPL [dB]) and vocal frequency (fundamental frequency [*F*0; Hz]) for females (*n* = 41) and males (*n* = 25).

Measures	NPNL vs. HPNL	NPNL vs. NPS	HPNL vs. NPS	
Normally distributed parameter				
	Post hoc: LSD (*p* ≤ .05)			ANOVA (*p* ≤ .05)
SPL (dB), female	*p* = .261	***p* < .001**	***p* < .001**	***F*(2, 120) = 32.01, *p* < .001**
SPL (dB), male	*p* = .420	***p* < .001**	***p* < .001**	***F*(2, 72) = 12.80, *p* < .001**
Nonnormally distributed parameter				
	Post hoc: Mann–Whitney *U* (*p* ≤ .05)			Kruskal–Wallis (*p* ≤ .05)
*F*0 (Hz), female	***U* = 1,593** ***p* < .001**	*U* = 1,070 *p* = .058	***U* = 1,540** ***p* < .001**	***H*(2) = 64.78** ***p* < .001**
*F*0 (Hz), male	***U* = 591** ***p* < .001**	*U* = 301.0 *p* = .831	***U* = 591** ***p* < .001**	***H*(2) = 39.21** ***p* < .001**

*Note.* Statistically significant values are in bold. LSD = least significant difference; ANOVA = analysis of variance.

**Table 4.** Mean values and standard deviations separated by gender and the three phonation tasks: normal pitch and soft (NPS), normal pitch and normal loudness (NPNL), and high pitch and normal loudness (HPNL).

Measures	Female group (*n* = 41)			Male group (*n* = 25)		
	NPNL	HPNL	NPS	NPNL	HPNL	NPS
Performed tasks (loudness and pitch)						
SPL (dB)	64.0 ± 5.3	65.2 ± 4.6	57.5 ± 3.9	61.8 ± 5.6	63.1 ± 7.3	55.3 ± 4.2
*F*0 (Hz)	231 ± 24	328 ± 58	225 ± 36	139 ± 20	223 ± 59	140 ± 23
Computed glottal area waveform measures						
Open quotient	0.93 ± 0.12	0.96 ± 0.10	0.96 ± 0.09	0.71 ± 0.19	0.88 ± 0.17	0.92 ± 0.13
Speed quotient	1.91 ± 0.59	1.69 ± 0.45	1.53 ± 0.34	1.42 ± 0.54	1.60 ± 0.56	1.12 ± 0.35
Rate quotient	2.24 ± 1.24	1.85 ± 0.67	1.67 ± 0.50	2.65 ± 1.24	2.15 ± 1.27	1.48 ± 0.90
Glottal gap index	0.07 ± 0.07	0.09 ± 0.08	0.11 ± 0.10	0.01 ± 0.01	0.05 ± 0.07	0.05 ± 0.07
Amplitude periodicity	0.98 ± 0.01	0.98 ± 0.01	0.98 ± 0.01	0.99 ± 0.00	0.98 ± 0.01	0.98 ± 0.01
Time periodicity	0.97 ± 0.01	0.96 ± 0.02	0.96 ± 0.01	0.98 ± 0.02	0.97 ± 0.02	0.97 ± 0.02
Phase asymmetry index	0.06 ± 0.04	0.08 ± 0.06	0.06 ± 0.04	0.06 ± 0.05	0.07 ± 0.05	0.07 ± 0.05
Spatial symmetry index	0.15 ± 0.08	0.13 ± 0.09	0.14 ± 0.09	0.17 ± 0.12	0.15 ± 0.11	0.15 ± 0.11
Amplitude symmetry index	0.80 ± 0.12	0.82 ± 0.11	0.81 ± 0.12	0.79 ± 0.13	0.81 ± 0.13	0.81 ± 0.12
Normalized maximum area declination rate	1.69 ± 0.44	1.39 ± 0.35	1.37 ± 0.40	1.64 ± 0.53	1.28 ± 0.51	1.25 ± 0.55
Amplitude-to-length ratio	11.95 ± 3.15	10.10 ± 3.10	11.25 ± 3.77	15.45 ± 4.74	10.20 ± 2.73	13.80 ± 3.91

*Note.* SPL = sound pressure level; *F*0 = fundamental frequency.

### Glottal Area Waveform Parameter Analysis Between Female and Male Participants

The impact of frequency and intensity on the glottal area waveform obtained from HSV recordings was quantified using the 11 kinematic variables computed from the glottal area waveform.

#### Glottal Area Waveform Changes Due to Frequency and Vocal Intensity Variations in Female Participants

Across the frequency and intensity conditions, the glottal area waveform measures of SQ, RQ, TP, MADRn, and ALR were statistically significant (see Table 5). Statistical significance was not obtained for OQ, GGI, AP, phase asymmetry index, spatial symmetry index, and amplitude symmetry index, suggesting that measures of glottal cycle periodicity and glottal cycle symmetry remained unaffected by variations in high-frequency and soft–vocal-intensity conditions in female participants (see Table 4).

- Effect of high frequency on the glottal area waveform: As expected, the MADRn was significantly smaller for the HPNL condition (*Mdn* = 1.33) compared with the NPNL condition (*Mdn* = 1.57). High-frequency phonation resulted in a significantly reduced value of the ALR (see Table 4) compared with the typical condition. In contrast, TP was significantly reduced for high-pitch phonation (HPNL [*Mdn* = 0.96], NPNL [*Mdn* = 0.97]) compared with the typical phonation.
- Effect of vocal intensity on the glottal area waveform: Post hoc analysis revealed that soft–vocal-intensity effects were observed on measures related to the glottal cycle quotients and glottal area waveform derivative measures. As seen in Table 4, measures of SQ (NPS [*Mdn* = 1.55], NPNL [*Mdn* = 1.89]), RQ (NPS [*Mdn* = 1.61], NPNL [*Mdn* = 2.09]), and MADRn (NPS [*Mdn* = 1.30], NPNL [*Mdn* = 1.57]) were significantly reduced for the soft (NPS) condition compared with the typical intensity (NPNL) condition.

**Table 5.** Statistical results for the female group (*n* = 41) across the three tasks: normal pitch and normal loudness (NPNL), high pitch and normal loudness (HPNL), and normal pitch and soft (NPS).

Glottal area waveform measures	NPNL vs. HPNL	NPNL vs. NPS	HPNL vs. NPS	
Normally distributed parameter				
	Post hoc: LSD (*p* ≤ .05)			ANOVA (*p* ≤ .05)
Amplitude-to-length ratio	***p* = .007, *d* = 0.569**	*p* = .307, *d* = 0.18	*p* = .091, *d* = 0.34	***F*(2, 120) = 3.97, *p* = .025**
Nonnormally distributed parameter				
	Post hoc: Mann–Whitney *U* (*p* ≤ .005)			Kruskal–Wallis (*p* ≤ .05)
Open quotient	—	—	—	*H*(2) = 3.67, *p* = .160
Speed quotient	*U* = 1,089 *p* = .026, *d* = 0.492	***U* = 1,321** ***p* < .001, *d* = 0.913**	*U* = 974 *p* = .112, *d* = 0.46	***H*(2) = 16.83, *p* < .001**
Rate quotient	*U* = 1,114 *p* = .011, *d* = −0.52	***U* = 1,292** ***p* < .001, *d* = 0.82**	*U* = 953.5 *p* = .209, *d* = 0.39	***H*(2) = 15.81, *p* < .001**
Glottal gap index	—	—	—	*H*(2) = 4.26, *p* = .119
Amplitude periodicity	—	—	—	*H*(2) = 4.34, *p* = .114
Time periodicity	***U* = 1,133.5** ***p* = .007, *d* = 0.73**	*U* = 847 *p* = .902, *d* = 0.03	***U* = 556.5** ***p* = .013, *d* = 0.67**	***H*(2) = 9.05, *p* = .011**
Phase asymmetry index	—	—	—	*H*(2) = 1.41, *p* = .493
Spatial symmetry index	—	—	—	*H*(2) = 0.63, *p* = .728
Amplitude symmetry index	—	—	—	*H*(2) = 0.733, *p* = .693
Normalized maximum area declination rate	***U* = 1,216** ***p* < .001, *d* = 0.683**	***U* = 1,329** ***p* < .001, *d* = 0.693**	*U* = 879 *p* = .581, *d* = 0.04	***H*(2) = 20.65, *p* < .001**

*Note.* Post hoc tests were performed for significant group findings. Bonferroni correction was applied on post hoc comparisons for the 10 nonnormally distributed measures (*p* ≤ .005 [.05/10]). Statistically significant values are in bold. Cohen's *d* effect sizes = *d* for significant variables. LSD = least significant difference; ANOVA = analysis of variance.

#### Glottal Area Waveform Changes Due to Frequency and Vocal Intensity Variations in Male Participants

Across the frequency and intensity conditions, the glottal area waveform measures of OQ, SQ, RQ, GGI, TP, MADRn, and ALR were statistically significant (see Table 6). Statistical significance was not obtained for AP, phase asymmetry index, spatial symmetry index, and amplitude symmetry index, across high-frequency and soft–vocal-intensity conditions for male participants (see Table 4).

- Effect of high frequency on the glottal area waveform: The values for the OQ (HPNL [*Mdn* = 0.95], NPNL [*Mdn* = 0.73]), and GGI (HPNL [*Mdn* = 0.02], NPNL [*Mdn* = 0.00]) were significantly higher for the high-frequency condition compared with the typical pitch condition. In contrast, TP (HPNL [*Mdn* = 0.97], NPNL [*Mdn* = 0.98]), MADRn (HPNL [*Mdn* = 1.25], NPNL [*Mdn* = 1.62]), and ALR (HPNL [*Mdn* = 10.55], NPNL [*Mdn* = 14.91]) were significantly reduced for the high-frequency condition compared with the typical condition.
- Effect of soft vocal intensity on the glottal area waveform: As seen in Table 3, glottal area waveform measures of OQ (NPS [*Mdn* = 0.98], NPNL [*Mdn* = 0.73]) and GGI (NPS [*Mdn* = 0.03], NPNL [*Mdn* = 0.00]) were significantly increased for the soft intensity condition compared with the typical intensity condition. In contrast, the values for RQ (NPS [*Mdn* = 1.28], NPNL [*Mdn* = 2.09]), TP (NPS [*Mdn* = 0.97], NPNL [*Mdn* = 0.98]), and MADRn (NPS [*Mdn* = 1.21], NPNL [*Mdn* = 1.62]) were significantly reduced for the NPS condition compared with the typical intensity condition.
- Combined effect of soft intensity and high frequency on the glottal area waveform: The SQ was significantly higher for the HPNL (*Mdn* = 1.5) condition compared with the NPS (*Mdn* = 1.19) condition. The RQ was significantly higher for the HPNL (*Mdn* = 1.73, *d* = 0.645) condition compared with the NPS (*Mdn* = 1.28) condition. In contrast, ALR was significantly reduced for the HPNL (*Mdn* = 10.55) condition compared with the NPS (*Mdn* = 13.23) condition.

**Table 6.** Statistical results for the male group (*n* = 25) across the three tasks: normal pitch and normal loudness (NPNL), high pitch and normal loudness (HPNL), and normal pitch and soft (NPS).

Glottal area waveform measures	NPNL vs. HPNL	NPNL vs. NPS	HPNL vs. NPS	
	Post hoc: Mann–Whitney *U* (*p* ≤ .004)			Kruskal–Wallis (*p* ≤ .05)
Open quotient	***U* = 484.5** ***p* < .001, *d* = −1.03**	***U* = 101.5** ***p* < .001, *d* = −1.4**	*U* = 275.5 *p* = .478, *d* = 0.32	***H*(2) = 19.10,** ***p* < .001**
Speed quotient	*U* = 381 *p* = .187, *d* = 0.36	*U* = 395 *p* = .112, *d* = 0.57	***U* = 466** ***p* = .003, *d* = 0.958**	***H*(2) = 8.86,** ***p* = .012**
Rate quotient	*U* = 206 *p* = .040, *d* = −0.43	***U = 555*** ***p* < .001, *d* = 1.126**	***U* = 471** ***p* = .002, *d* = 0.645**	***H*(2) = 24.03,** ***p* < .001**
Glottal gap index	***U* = 466** ***p* = .002, *d* = −0.83**	***U* = 115.5** ***p* < .001, *d* = −0.914**	*U* = 287 *p* = .626, *d* = 0.03	***H*(2) = 16.67,** ***p* = .001**
Amplitude periodicity	—	—	—	*H*(2) = 5.35, *p* = .069
Time periodicity	***U* = 123.5** ***p* < .001, *d* = 0.67**	***U* = 496** ***p* < .001, *d* = 0.611**	*U* = 313 *p* = .99, *d* = 0.17	***H*(2) = 17.53,** ***p* = .001**
Phase asymmetry index	—	—	—	*H*(2) = 2.21, *p* = .331
Spatial symmetry index	—	—	—	*H*(2) = 0.49, *p* = .781
Amplitude symmetry index	—	—	—	*H*(2) = 0.58, *p* = .746
Normalized maximum area declination rate	***U* = 145** ***p* = .001, *d* = 1.064**	***U* = 519** ***p* < .001, *d* = 1.284**	*U* = 319 *p* = .907, *d* = 0.14	***H*(2) = 17.78,** ***p* < .001**
Amplitude-to-length ratio	***U* = 82** ***p* < .001, *d* = 1.449**	*U* = 384 *p* = .168, *d* = 0.41	***U* = 124** ***p* < .001, *d* = −1.199**	***H*(2) = 23.62,** ***p* < .001**

*Note.* Post hoc tests were performed for significant group findings. Bonferroni correction was applied on post hoc comparisons (*p* ≤ .004 [.05/11]) for all 11 nonnormally distributed measures. Statistically significant values are in bold. Cohen's *d* effect size = *d* for significant variables.

### Strategies for Frequency and Vocal Intensity Variations

**Figure 1.**
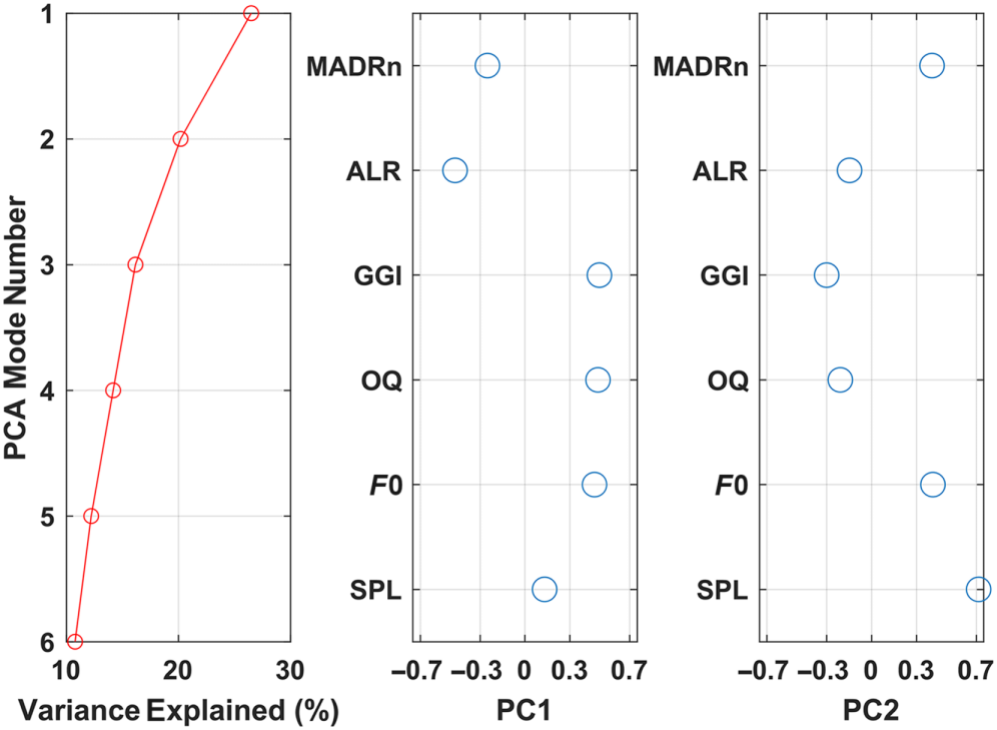
The percentage of variance explained by each principal component analysis (PCA) mode and the contribution (loading) of individual glottal area waveform measures to the first two PCA modes. The analysis included two control measures, namely, fundamental frequency (*F*0) and vocal sound pressure level (dB SPL), along with four glottal area waveform measures, namely, normalized maximum area declination rate (MADRn), amplitude-to-length ratio (ALR), glottal gap index (GGI), and open quotient (OQ), allowing us to relate changes in glottal area waveform measures to changes in the target SPL and *F*0. In the first principal component (PC1), *F*0 shows a substantially higher loading (0.46) than SPL (0.13), indicating that PC1 primarily reflects a pitch control strategy. In contrast, in the second principal component (PC2), SPL has a higher loading (0.72) than *F*0 (0.41), suggesting that PC2 primarily reflects a vocal intensity control strategy.

The results from the PCA are shown in Figure 1. The first two principal components captured 26.5% and 20.2% of the total variance, respectively. In the first principal component (PC1), *F*0 has a much larger loading (0.46) than the SPL (0.13), indicating that PC1 describes primarily a pitch control strategy. Specifically, an increase in *F*0 is accompanied by an increased degree of glottal opening (increased minimal glottal area and OQ), a decreased ALR, and less skewed glottal area waveform (reduced MADRn). The simultaneous increase in the OQ and *F*0 suggests that PC1 is more associated with increasing activation of the cricothyroid muscles rather than increasing vocal fold adduction. In contrast, for the second principal component (PC2), the SPL has a much larger loading (0.72) than *F*0 (0.41), suggesting that PC2 describes primarily a vocal-intensity control strategy. Specifically, a reduction in vocal intensity is accompanied by a decreased *F*0, decreased skewedness of the glottal area waveform (increased MADRn), increased minimal glottal area (GGI), increased OQ, and increased ALR. Since the OQ and MADRn are primarily controlled by vocal fold thickness (Zhang, 2016b, 2024b), this suggests that PC2 corresponds to a strategy of decreasing vocal intensity through reduced vocal fold adduction, likely coordinated with a reduction in subglottal pressure.

**Figure 2.**
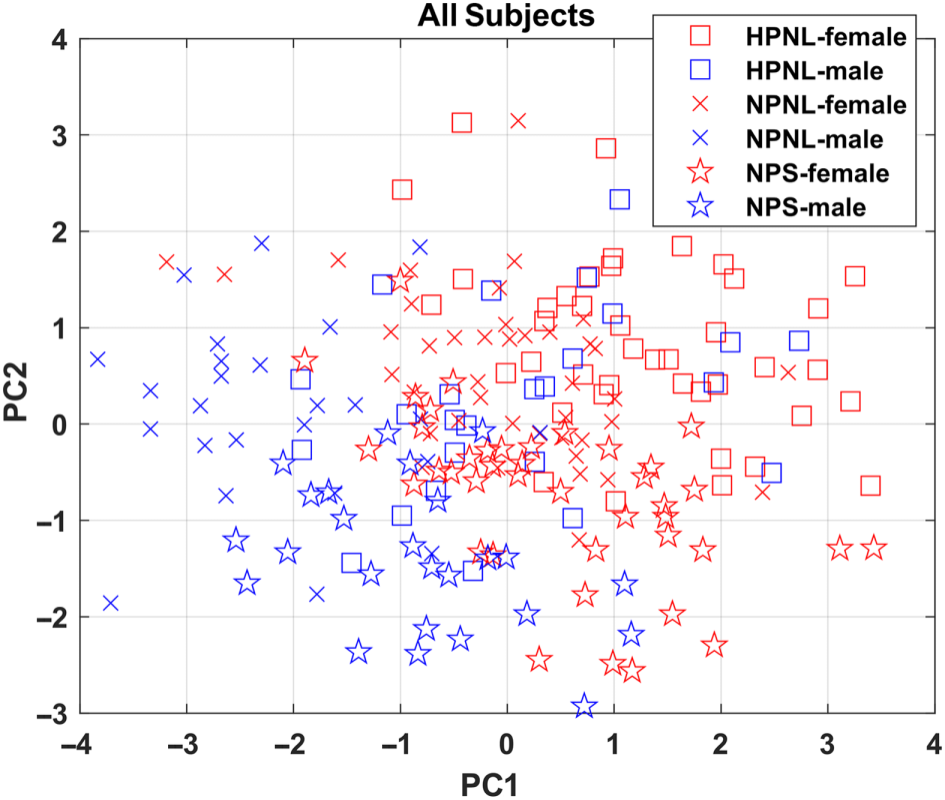
Principal component analysis (PCA) showing clustering patterns among all participants and among the six groups differentiated by sex and condition: high-pitch normal loudness (HPNL), normal pitch and normal loudness (NPNL), and normal pitch and soft (NPS). Red and blue markers represent female and male participants, respectively, with distinct shapes for each group: squares for HPNL, crosses for NPNL, and stars for NPS, highlighting group-specific distributions along PCA Components 1 and 2 (PC1 and PC2, respectively).

Figure 2 shows that the female and male participants occupied different regions in the PC1–PC2 space, mean difference = 1.55, *SE* = 0.20, *t*(138) = 7.72, *p* < .001, for PC1, and mean difference = 0.57, *SE* = 0.17, *t*(151) = 3.42, *p* < .001, for PC2, with the male participants having a more negative value in the coefficient along the PC1 axis. This indicates that the female participants tended to have lower values in ALR and higher values in *F*0, OQ, and GGI, as shown in Table 4.

**Figure 3.**
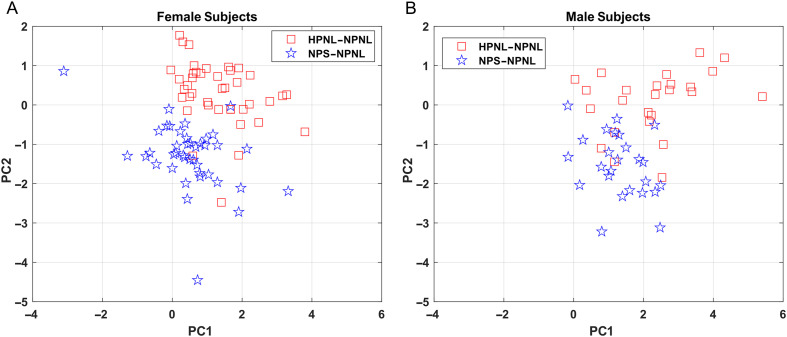
Adjustments by each female (A) and male (B) participant in the space spanned by the first two principal components (PC1 and PC2) when changing from typical pitch and loudness (normal pitch and normal loudness [NPNL]) to increased pitch (high pitch and normal loudness [HPNL]–NPNL) or reduced vocal intensity (normal pitch and soft [NPS]–NPNL). Each symbol represents the difference in values from HPNL and NPNL (red squares) and NPS and NPNL (blue stars).

Figures 3A and 3B show changes in the coefficients of PC1 and PC2 within each participant when they increased pitch (HPNL–NPNL) or reduced loudness (NPS–NPNL), for both the female (see Figure 3A) and male (see Figure 3B) participants. While large interspeaker variability can be observed, most participants increased pitch by adjustments mainly along PC1 (increased cricothyroid muscle activation), with a mean difference of 1.31, *SE* = 0.15, *t*(40) = 9.01 (*p* < .001), for female participants, and a mean difference of 2.25, *SE* = 0.26, *t*(24) = 8.52 (*p* < .001), for male participants, and the changes in PC2 were not significantly different from zero, with a mean difference of 0.30, *SE* = 0.12, *t*(40) = 2.38 (*p* = .022), for female participants, and a mean difference of 0.08, *SE* = 0.16, *t*(24) = 0.53 (*p* = .603), for male participants. From typical phonation to soft phonation, most participants adjusted along both the PC2 (reduced vocal fold adduction) and PC1 (increased cricothyroid muscle activation), with changes in PC1 and PC2 both significantly differently from zero (except for female participants in which changes in PC1 were marginally statistically significant). For PC1, from typical phonation to soft phonation, the mean difference was 0.49, *SE* = 0.16, *t*(40) = 3.07 (*p* = .004), for female participants, and the mean difference was 1.31, *SE* = 0.15, *t*(24) = 8.47 (*p* < .001), for male participants. For PC2, the mean difference was −1.28, *SE* = 0.13, *t*(40) = −9.76 (*p* < .001), for female participants, and the mean difference was −1.52, *SE* = 0.16, *t*(24) = −9.39 (*p* < .001), for male participants.

## Discussion

The purpose of this study was to establish quantitative group data for various measures of the glottal area waveform as a function of pitch and loudness in vocally healthy adult male and female participants. Vocal function was quantified using glottal cycle quotients, periodicity, symmetry, and glottal area waveform derivatives of MADRn and ALR. Prior research on the physiological changes associated with frequency and vocal intensity has primarily relied on theoretical/computational models (Titze, 1988, 1989a; Titze & Hunter, 2004; Titze & Sundberg, 1992; Yin & Zhang, 2014; Zhang, 2016a, 2016b, 2016c, 2024a), in vivo animal models (Luegmair et al., 2014; Vahabzadeh-Hagh et al., 2017), and excised larynx models (Tanaka & Tanabe, 1986; Zhang & Chhetri, 2019). Aerodynamic investigations, by Holmberg and colleagues (Holmberg et al., 1988, 1989), have also contributed to understanding the relationships between subglottal pressure, airflow, and acoustic output, across different vocal frequencies and intensities. However, direct measurements of the HSV-derived glottal area waveform in human participants remain limited, especially across high frequency and soft vocal intensity, as most studies have been on modal/typical phonation (Ahmad et al., 2012; Arias-Vergara et al., 2023; Bohr et al., 2013; R. R. Patel et al., 2014a; Schlegel et al., 2020). While some studies have used videostroboscopy to quantify vocal fold vibration as a function of frequency and vocal intensity (Woo, 1996), these methods lack the temporal resolution and precision offered by more advanced techniques, such as HSV, and thereby limit its effectiveness in advancing clinical and theoretical understanding of vocal function and disorders.

### Glottal Area Waveform Changes Due to High-Frequency Phonation

**Figure 4.**
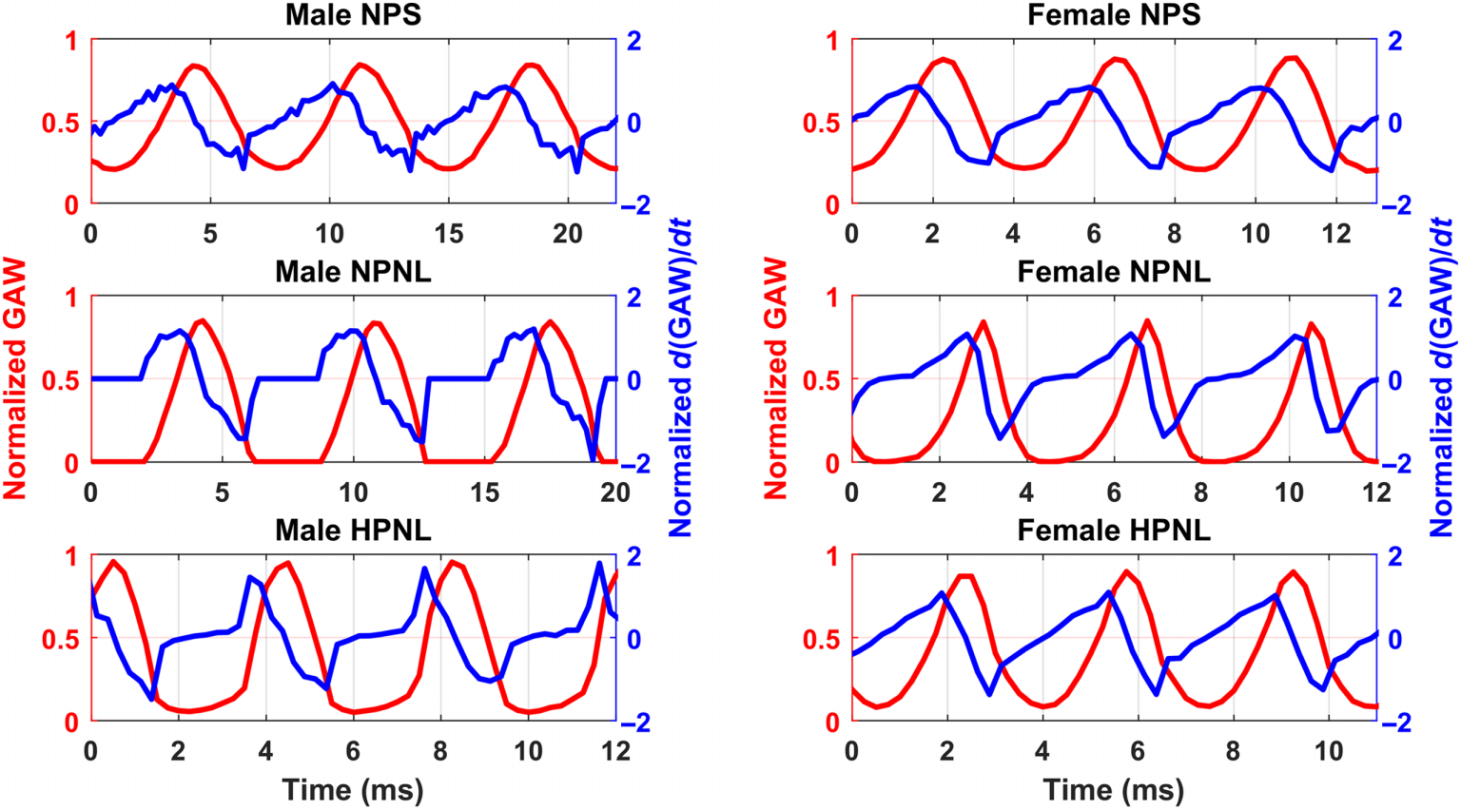
Representative normalized glottal area waveform (GAW) and its time derivative (*d*[GAW]/*dt*) during soft vocal intensity (normal pitch and soft [NPS]), typical phonation (normal pitch and normal loudness [NPNL]), and high-frequency phonation (high pitch and normal loudness [HPNL]) for one male and female speaker. Points where *d*(GAW)/*dt* = 0 indicate that the glottal opening is at either maximum or minimum; positive values indicate that the glottis is opening (increasing area), and negative values indicate that it is closing (decreasing area).

In both female and male participants, high-frequency phonation resulted in systematic changes in several glottal area waveform measures that are consistent with increased activation of the cricothyroid muscles. These include a smoother glottal area waveform, an increased minimal glottal opening, and an increased OQ, suggesting reduced vocal fold thickness and approximation (see Figure 4). High-frequency phonation also resulted in a decreased ALR. The observed increase in glottal opening and reduction in amplitude align with findings from Rasp et al. (2006), who reported increased glottal gap based on visual–perceptual analysis and decreased amplitude from HSV-derived trajectories in 29 female participants as pitch increased. However, the present findings are based on quantitative measures from the glottal area waveform. Considering that the SPL slightly increased in high-pitch phonation, the reduced ALR suggests an increase in vocal fold longitudinal tension and stiffness, which reduces vibration amplitude and possibly an elongated vocal fold. Overall, this suggests that high-frequency phonation in most participants was achieved through an increased activity of the cricothyroid muscles, as supported by the results from PCA. The simultaneous increase in the OQ and *F*0 suggests that PC1 is more associated with increasing activation of the cricothyroid muscles rather than increasing vocal fold adduction, as discussed in the introduction section. This is consistent with the finding of an increase in length with increasing pitch in six male participants (Hollien & Moore, 1960).

Some differences were observed between male and female participants. For example, while the OQ and GGI were increased in all participants, this increase was significant only in male participants. This may be partially due to the relatively higher baseline values in female than male participants, which makes the relative magnitude of changes smaller. Moreover, the OQ is calculated based on the absolute glottal area, which may not be the most accurate method due to the presence of a persistent glottal gap (Kist et al., 2021). Overall, the MADRn values were slightly lower in male compared with female participants. Zhang (2024b) shows that MADRn increases with increasing thickness but decreases with increasing length. Thus, this difference in MADRn between female and male participants suggests that the length effect slightly outweighs the thickness effect in male participants.

The findings of this study align with previous research on typical phonation and glottal cycle quotients (Bohr et al., 2013; R. R. Patel et al., 2014b, 2021). Similar to Kitzing and Sonesson (1974), we did see a trend of an increase in OQ and a decrease in SQ and RQ. Their study, which included 20 female participants, was primarily qualitative and did not include statistical analysis. In contrast, we conducted a statistical analysis of these measures; however, the differences were only statistically significant in male participants, as discussed earlier. The finding of increased OQ aligns with the high-speed film studies by Timcke et al. (1958), which observed only slight changes in OQ with pitch. These findings align with previous observations of increased glottal airflow at higher pitches in male participants (Holmberg et al., 1989). Although absolute vibrational amplitude was not measured here, our study provides first empirical evidence that the ALR in male participants is approximately 1.29 times that in female participants—a scale factor similar to the value of 1.2 reported in a previous computational study (Titze, 1989b). However, our finding of reduced TP is contradictory to the notion that increased vocal frequency tends to result in a more stable *F*0 with decreased jitter and increased motor-unit firing rate. (Titze, 1991).

The identification of two principal components from the PCA provided important insights into the potential strategies used by speakers to achieve the intended changes in target *F*0 and vocal intensity. However, these two principal components captured only about 47% of the total variance, indicating considerable variability in speaker-specific strategies in modulating voice production, which is worth further investigation.

### Glottal Area Waveform Changes Due to Soft Phonation

Soft phonation resulted in notable alterations in the glottal area waveform measures that are mostly consistent with reduced/no vocal fold adduction and/or reduced subglottal pressure, as well as slight compensatory cricothyroid muscle activation. These include reduced SQ and MADRn as well as increased OQ and GGI. This indicates vocal fold vibration with incomplete glottal closure, a reduced duration of glottal closure, and a more symmetric glottal area waveform (see Figure 4), often produced by a reduced vocal fold thickness and approximation (Zhang, 2021a, 2024b). The PCA further shows that most participants reduced vocal fold adduction (PC2) with simultaneously increased cricothyroid muscle activity (PC1), likely in an effort to maintain the same pitch target, particularly in female participants. Since adjustments along PC1 and PC2 have an opposite effect on the ALR, this difference also explains why soft phonation was not accompanied by significant changes in the ALR. Similar to the effects observed in high-pitch phonation, soft phonation resulted in an increase in OQ and GGI, which were statistically significant only in male participants. This may be related to the relatively high baseline values in OQ and GGI in female participants, which makes the relative magnitude of changes smaller.

The findings of reduced RQ and SQ for soft voice are similar to those reported by Kitzing and Sonesson (1974) in 20 female participants. Reduced SQ was also noted in R. R. Patel et al.'s (2022) study from six female participants and seven male participants. The increase in OQ when producing soft phonation aligns with the high-speed film studies by Timcke et al. (1958), which reported an inverse relationship of OQ with loudness. An increase in GGI is similar to findings reported using videostroboscopy (Södersten & Lindestad, 1990; Sulter & Albers, 1996). Soft vocal intensity is often accompanied by lower pressure, increased airflow, and lower vocal aerodynamic efficiency (Holmberg et al., 1988; Isshiki & Vonleden, 1964; R. R. Patel et al., 2022; Stathopoulos & Sapienza, 1993). Our in vivo findings are consistent with results from ex vivo synthetic models, which demonstrated lower biomechanical efficiency with reduced vocal intensity, decreased subglottal pressure, and smaller absolute maximum area declination rate in cases of glottal closure insufficiency (Kniesburges et al., 2020).

Interestingly, when comparing vocal frequency and intensity effects together in male participants, the RQ was significantly higher in the HPNL condition compared with soft phonation, while ALR was reduced. This points to distinct and sometimes opposing contributions of vocal frequency and intensity to the glottal area waveform structure, with vocal frequency tending to preserve or increase certain asymmetries, while soft vocal intensity dampens them.

### Additional Methodological Considerations

Although this prospective study provides important empirical data on glottal area waveform measures across vocal frequency and intensity conditions in vocally healthy adults, several limitations should be considered. First, the sample consisted exclusively of healthy young adult male and female speakers, which limits generalizability across different age groups. Second, this study was not designed to examine sex effects; future studies with larger, sex-balanced samples are therefore needed to more systematically investigate these differences. Third, a limitation of this study is that vocal frequency and SPL were self-selected across the tasks, which may introduce interspeaker variability and may have some effect on the physiological measures; however, this approach was chosen to preserve ecological validity. Furthermore, our task performance data show that participants appropriately differentiated among the three tasks (see Table 3), producing typical, high-pitch, and soft phonation as instructed. Fourth, the two-dimensional nature of HSV precludes the direct measurement of vocal fold vertical displacement, mucosal wave propagation in the medial–lateral dimension, and true vibratory amplitude. To overcome these spatial and dimensional constraints, future work would benefit from the integration of three-dimensional laser imaging techniques, which can provide real-time three-dimensional reconstruction of the vocal fold medial surface during phonation (R. R. Patel, Döllinger, Jakubass, et al., 2024; R. R. Patel, Döllinger, & Semmler, 2024; Semmler et al., 2016, 2018). Finally, derivation of physiological inferences from glottal area waveform measures such as MADRn and ALR rests on assumptions regarding the relationship between surface kinematics and underlying tissue biomechanics. Without direct physiological measures (e.g., electromyography for cricothyroid or thyroarytenoid, subglottal pressure monitoring), interpretations of neuromuscular strategies remain inferential, albeit supported by previous modeling and experimental work. Future studies incorporating simultaneous aerodynamic, acoustic, and neuromuscular data would help strengthen the mechanistic interpretations of glottal area waveform–derived measures.

## Conclusions

This study presents normative data for multidimensional measures of the glottal area waveform in vocally healthy adults producing sustained /i:/ with typical-frequency and vocal-intensity, high-frequency, and soft–vocal-intensity phonations. The results showed that the measures of SQ, RQ, MADRn, and ALR appear to be more salient capturing the differences as a result of increased frequency and reduced vocal intensity compared with the other measures. Despite considerable interspeaker variability, most participants increased pitch primarily along PC1, consistent with increased cricothyroid muscle activation, whereas changes along PC2 were not significant, indicating minimal modulation of vocal fold adduction during pitch elevation. In contrast, during soft phonation, participants adjusted along both PC1 and PC2, reflecting combined effects of reduced vocal fold adduction and increased cricothyroid activation, leading to glottal area waveform changes that reflect more incomplete closure and a less skewed glottal area waveform—patterns more prominent in male participants due to baseline sex differences. These patterns suggest that participants predominantly rely on cricothyroid muscle adjustments to modulate pitch, whereas soft phonation involves coordinated changes in both glottal adduction and cricothyroid activation, highlighting task-dependent strategies in glottal control that are measurable in the glottal area waveform. Clinically, this suggests that voice therapy and assessment should account for these physiological strategies and baseline sex differences, particularly when targeting pitch control, glottal closure, or vocal efficiency in voice disorders.

In an effort to move toward generalizable norms, future studies should concentrate on significant measures, to develop a large-scale normative database across the life span. It remains to be determined if older adults with presbyphonia or prepubertal children use the same strategies to achieve variations in vocal frequency and intensity. The current study provides normative data for the comparison of young adults with dysphonia.

## Data Availability Statement

Data are available from the corresponding author upon request.
